# Phenotype-Specific Pharmacogenetic Patterns of Methotrexate Neurotoxicity in Pediatric Acute Lymphoblastic Leukemia

**DOI:** 10.3390/jcm15176877

**Published:** 2026-09-05

**Authors:** Javier Gómez-Román, Juan C. Restrepo, Luz M. González, Laura González-Rodríguez, Ángela Lacombe-Antoneli, Yolanda Gutiérrez-Martín, María Dolores de la Maya, Montserrat Mesegué, José Luis Dapena, Ana Carbone, Berta González Martínez, Francisco Lendínez Molinos, Antonio Molinés Honrubia, Miriam Abós García, Marina García Morín, Samuel Navarro Noguera, María Sagaseta de Ilurdoz, Itziar Astigarraga, Maria Baro Fernández, Jaime Verdú Amorós, Alexandra Regueiro, Manuel Ramírez Orellana, José Luis Fuster Soler, Soledad González Muñiz, Adela Cañete Nieto, Montserrat Torrent Español, Héctor González Méndez, Jose M. Vagace, Guillermo Gervasini

**Affiliations:** 1 Department of Medical & Surgical Therapeutics, School of Medicine, University of Extremadura, 06006 Badajoz, Spain; jgomezroman@laboratorioslarrasa.com (J.G.-R.); luzmariagg@unex.es (L.M.G.); lgonzalezn@alumnos.unex.es (L.G.-R.); 2 Laboratorios Larrasa Human Personalized Medicine, 06170 Badajoz, Spain; aantoneli@laboratorioslarrasa.com; 3 Catalan Institute of Oncology, 43005 Tarragona, Spain; juan.restrepocorrea@gmail.com; 4 Bioscience Applied Techniques Services, Servicio de Apoyo a la Investigación, University of Extremadura, 06006 Badajoz, Spain; ygmartin@unex.es; 5 Service of Hematology, Materno-Infantil Hospital, 06010 Badajoz, Spain; delamayaretamar@yahoo.es; 6 Service of Pediatric Hematology and Oncology, Sant Joan de Déu Barcelona Children’s Hospital, 08950 Barcelona, Spain; mmesegue@sjdhospitalbarcelona.org (M.M.); jdapena@sjdhospitalbarcelona.org (J.L.D.); 7 Service of Pediatric Hematology and Oncology, Miguel Servet University Hospital, 50009 Zaragoza, Spain; acarboneb@gmail.com; 8 Service of Pediatric Hematology and Oncology, La Paz University Hospital, 28046 Madrid, Spain; berta.gonzalez@salud.madrid.org; 9 Service of Pediatric Hematology and Oncology, Princesa Leonor Materno Infantil Hospital, 04009 Almería, Spain; lendimol@gmail.com; 10 Service of Pediatric Hematology and Oncology, Complejo Hospitalario Universitario Insular-Materno Infantil, 35016 Las Palmas de Gran Canaria, Spain; amolhon@gobiernodecanarias.org; 11 Service of Pediatric Hematology and Oncology, Donostia University Hospital, 20014 San Sebastián, Spain; miriam.garciaabos@osakidetza.eus; 12 Service of Pediatric Hematology and Oncology, Gregorio Marañón General University Hospital, 28007 Madrid, Spain; mgmorin@salud.madrid.org; 13 Service of Pediatric Hematology and Oncology, Son Espases University Hospital, 07120 Palma, Spain; samuel.navarro@ssib.es; 14 Service of Pediatric Hematology and Oncology, Virgen del Camino Hospital, 31008 Pamplona, Spain; mariasagaseta8361@gmail.com; 15 Service of Pediatric Hematology and Oncology, Cruces University Hospital, 48903 Barakaldo, Spain; itziar.astigarraga@osakidetza.net; 16 Service of Pediatric Hematology and Oncology, 12 de Octubre University Hospital, 28041 Madrid, Spain; maria.baro@salud.madrid.org; 17 Service of Pediatric Hematology and Oncology, Hospital Clínico Universitario de Valencia, 46010 Valencia, Spain; verdu_josamo@gva.es; 18 Service of Pediatric Hematology and Oncology, University Clinical Hospital of Santiago de Compostela, 15706 Santiago de Compostela, Spain; alexandra.regueiro.garcia@sergas.es; 19 Service of Pediatric Hematology and Oncology, Niño Jesús University Children’s Hospital, 28009 Madrid, Spain; manuel.ramirez@salud.madrid.org; 20 Service of Pediatric Hematology and Oncology, Virgen de la Arrixaca University Clinical Hospital, 30120 Murcia, Spain; josel.fuster@um.es; 21 Service of Pediatric Hematology and Oncology, Central University Hospital of Asturias, 33011 Oviedo, Spain; soledad.gonzalezm@sespa.es; 22 Service of Pediatric Hematology and Oncology, La Fe University and Polytechnic Hospital, 46026 Valencia, Spain; adela.canete@uv.es; 23 Service of Pediatric Hematology and Oncology, Hospital de la Santa Creu i Sant Pau, 08041 Barcelona, Spain; mtorrent@santpau.cat; 24 Service of Pediatric Hematology and Oncology, University Hospital of the Canary Islands, 38320 San Cristóbal de La Laguna, Spain; hgonmen@gobiernodecanarias.org; 25 Department of Biomedical Sciences, School of Medicine, University of Extremadura, 06006 Badajoz, Spain; 26 Institute of Molecular Pathology Biomarkers, University of Extremadura, 06006 Badajoz, Spain

**Keywords:** acute lymphoblastic leukemia, methotrexate, neurotoxicity, next-generation sequencing, pharmacogenomics

## Abstract

**Background:** Methotrexate-induced neurotoxicity (MTX-NTX) is a severe complication during childhood acute lymphoblastic leukemia (ALL) treatment, yet its underlying pharmacogenetic determinants remain incompletely understood. **Methods:** A multicenter retrospective nationwide case–control study was performed including 71 pediatric ALL patients (34 MTX-NTX cases and 37 controls). Targeted next-generation sequencing of 17 genes involved in MTX transport, intracellular metabolism and folate pathways was performed. **Results:** Transport-related genes accounted for 61.4% of all detected variants and showed the highest number of nominally significant variants. Pathway-level burden analysis demonstrated a significant enrichment of transporter-related variants in patients with MTX-NTX compared with controls (FDR-adjusted *p* = 0.004), whereas the analysis for folate/metabolism-related genes did not remain significant after multiple-testing (FDR-adjusted *p* = 0.096). Similar findings were observed in the stroke-like syndrome (SLS) subgroup (FDR-adjusted *p* = 0.014 and 0.108, respectively). Gene-level burden analysis identified *ABCG2* and *ABCC4* as the predominant contributors, harboring both 18.4% of significant variants in the overall MTX-NTX analysis and 20.6% and 17.6%, respectively in the SLS phenotype. Moreover, the distribution of significant variants was strongly correlated between the overall MTX-NTX and SLS analyses (Spearman’s ρ = 0.549, *p* = 0.022). Volcano plot analysis revealed *ABCG2* as the gene showing the most prominent pattern of nominal associations, containing both the variants associated with a protective direction (e.g., c.1728-46G>A, *p* = 2.6 × 10^−4^) and variants associated with increased risk, including c.34G>A and c.203+36A>G (*p* = 0.002) and c.263+10A>G (*p* = 0.0097). Phenotype-specific analyses revealed distinct genetic signatures across neurological manifestations, with transporter genes predominating in focal neurological deficits. **Conclusions:** Our findings suggest that genetic variability in ABC transporter genes may contribute to susceptibility to MTX-NTX, whereas folate metabolism genes may represent complementary phenotype-modifying factors. These findings are exploratory and require validation in larger, independent cohorts and functional studies.

## 1. Introduction

Acute lymphoblastic leukemia (ALL) is the most common pediatric malignancy and currently represents one of the greatest success stories in pediatric oncology, with survival rates exceeding 90% in high-income countries [1,2]. As survival outcomes continue to improve, current therapeutic strategies increasingly focus on reducing treatment-related toxicity and long-term sequelae without compromising antileukemic efficacy. Among these complications, neurotoxicity (NTX) remains one of the most clinically relevant adverse events in children receiving chemotherapy for ALL, as it may lead to treatment interruptions, prolonged hospitalization, long-term neurocognitive impairment, and, in severe cases, permanent neurological deficits or death [3,4,5]. NTX is a complex, multifactorial phenotype resulting from the interaction between treatment-related, clinical, and genetic factors rather than from the effect of a single genetic variant. Furthermore, survivors of childhood ALL exposed to neurotoxic chemotherapy may develop persistent deficits in executive functioning, memory, processing speed, and quality of life years after treatment completion [3,4].

Methotrexate (MTX) is the cornerstone of modern ALL treatment protocols and is administered through both intrathecal (IT) and high-dose intravenous (IV) schedules to prevent and treat central nervous system (CNS) leukemia involvement. Despite its essential therapeutic role, MTX is associated with a broad spectrum of neurological adverse events collectively referred to as MTX-induced neurotoxicity (MTX-NTX) [6,7], with a reported incidence of 1–15% [8,9,10,11].

These complications are clinically heterogeneous and may present as acute, subacute, or delayed neurological syndromes [12]. The most frequently described phenotypes include stroke-like syndrome (SLS), posterior reversible encephalopathy syndrome (PRES), and isolated seizures [9,10,13]. This complex genetic architecture provides a rationale for the use of targeted next-generation sequencing (NGS) approaches, which allow the simultaneous assessment of multiple candidate genes and variants within biologically relevant pathways.

The basis of MTX metabolism involves MTX transport, intracellular disposition, and folate metabolism. The transporter component included the ATP-binding cassette (ABC) efflux transporters *ABCB1*, *ABCC1*-*5*, and *ABCG2*, as well as the solute carrier transporters *SLC19A1*, *SLC22A6*, and *SLC22A8*, which are involved in cellular uptake, transport, or efflux of methotrexate and related metabolites. The folate and intracellular MTX metabolism component included *MTHFR*, *MTR*, *MTRR*, *TYMS*, *ATIC*, and *FPGS*, representing key steps in folate homeostasis, one-carbon metabolism, thymidylate synthesis, purine metabolism, and intracellular methotrexate polyglutamation. *COL18A1* was also included as a candidate gene based on its potential relevance to vascular and endothelial mechanisms that may contribute to the pathophysiology of methotrexate-associated neurological toxicity [14]. This panel-based strategy therefore enabled the simultaneous assessment of genetic variation across complementary biological pathways potentially involved in MTX-NTX. In recent years, increasing evidence has suggested that host genetic factors may also contribute significantly to the development of MTX-NTX. Previous pharmacogenetic studies of MTX toxicity in childhood ALL have pointed out variants in genes involved in folate intracellular pathways and MTX transport as potential modifiers of MTX pharmacokinetics (PK) and neurotoxicity susceptibility [15,16,17]. Genome-wide association studies of MTX-related neurotoxicity have further identified susceptibility loci involving not only pharmacokinetic pathways but also genes related to neuronal development and cytoskeletal organization [4,9]. More recently, high-throughput sequencing approaches have expanded this landscape: integrated genomic analyses including whole-exome sequencing have identified determinants of intracellular MTX polyglutamate accumulation, including MTX transporters such as *ABCC1* and *ABCC4* [18], targeted NGS studies have characterized broader variation across ABC and SLC transporter genes [19] and whole-exome sequencing has identified novel variants associated with delayed HD-MTX clearance in pediatric ALL [20]. Nevertheless, available data remain limited and sometimes contradictory, largely due to heterogeneous definitions of NTX and differences in study designs [9,17], and recent studies have shown only modest predictive improvement when previously reported MTX pharmacogenomic variants are added to clinical risk factors [21].

Therefore, the aim of this multicenter, nationwide study was to investigate the potential contribution of genetic variants in MTX transport and metabolic pathways to the clinical manifestations and phenotype-specific presentation of MTX-NTX in pediatric ALL. Using targeted NGS of 17 candidate genes, we sought to identify genetic patterns associated with susceptibility to MTX-NTX and its distinct clinical phenotypes. In addition, given the relatively low incidence of both childhood ALL and MTX-NTX, recruitment at a nationwide level was essential to assemble a sufficiently informative cohort for this exploratory pharmacogenetic investigation. Nevertheless, the sample size limits the statistical power to detect associations involving rare variants and therefore the findings should be considered hypothesis-generating and require validation in larger independent cohorts.

## 2. Materials and Methods

### 2.1. Study Design and Patient Cohort

A retrospective multicenter nationwide study was conducted including pediatric patients diagnosed with ALL in Spain who received MTX-based treatment SHOP protocols from the Spanish Society of Pediatric Hematology and Oncology (SEHOP) between 2003 and 2022. The study was carried out in the frame of the Leukemia Working Group of SEHOP. The study population consisted predominantly of pediatric patients of Spanish origin recruited from multiple centers across Spain. Demographic information, including self-reported ethnicity, was collected from the clinical records. Patients who developed MTX-NTX (*n* = 34) were identified according to the criteria published by Inaba et al. [22] and compared with a control cohort of randomly selected children with ALL, who were treated according to the same protocols, received the same doses of MTX but did not develop neurological complications (*n* = 37). Given the limited number of MTX-NTX events and the differences in age observed between cases and controls, potential confounding was addressed in the genetic association analyses using Firth’s penalized logistic regression, incorporating age and sex as covariates.

Inclusion criteria: Patients were eligible for inclusion if they were younger than 18 years at the time of ALL diagnosis. Patients with incomplete clinical information or unavailable/incomplete brain magnetic resonance imaging (MRI) data required for the assessment and classification of MTX-NTX were excluded from the final analysis.

Neurotoxicity phenotypes were classified according to previously described clinical and radiological criteria, including SLS, PRES, MTX-NTX not otherwise specified (MTX-NTX NOS), and isolated seizures [12]. SLS is characterized by transient focal neurological deficits, commonly including hemiparesis, dysarthria, aphasia, or sensory disturbances, frequently accompanied by seizures and characteristic diffusion restriction on magnetic resonance imaging (MRI) [4,7]. PRES, in contrast, typically presents with headache, altered mental status, seizures, visual disturbances, and vasogenic edema predominantly involving posterior brain regions [13]. The term MTX-NTX NOS refers to patients who developed symptoms of central NTX following the administration of IV or IT MTX but who did not meet sufficient criteria to be classified as any of the clinical–radiological phenotypes described above.

All patients were treated according to standard protocols from SEHOP active at their time of diagnosis: SHOP 99, SHOP 05, and SEHOP-PETHEMA 13. These protocols comprise induction, consolidation, and maintenance phases, with specific intensification blocks for high-risk patients. Standard planned IT MTX doses were 10 doses for SHOP 99 and SHOP 05 and 15 doses for SEHOP-PETHEMA 13 (increasing to 14, 14, and 17 doses for high-risk patients, respectively). Total planned IV MTX doses were 12 g/m^2^ for SHOP 99 and 15 g/m^2^ for both SHOP 05 and SEHOP-PETHEMA 13 (increasing to 28, 25, and 40 g/m^2^ for high-risk patients, respectively). Cumulative IV MTX dose (g/m^2^) and total number of IT MTX administrations received were recorded for all subjects. Additionally, the route of administration immediately preceding the neurotoxic event and the corresponding treatment phase were documented.

Written informed consent was obtained from patients and/or legal guardians in accordance with institutional guidelines. The study was approved by the corresponding institutional ethics committee (No 56/2016) and conducted in accordance with the Declaration of Helsinki and its subsequent amendments.

During the preparation of this manuscript, the authors used ChatGPT (GPT-5.6 Luna) for the purposes of improving its readability and language. The authors have reviewed and edited the output and take full responsibility for the content of this publication.

### 2.2. Targeted Sequencing Panel and MTX Pathway

**Figure 1:**
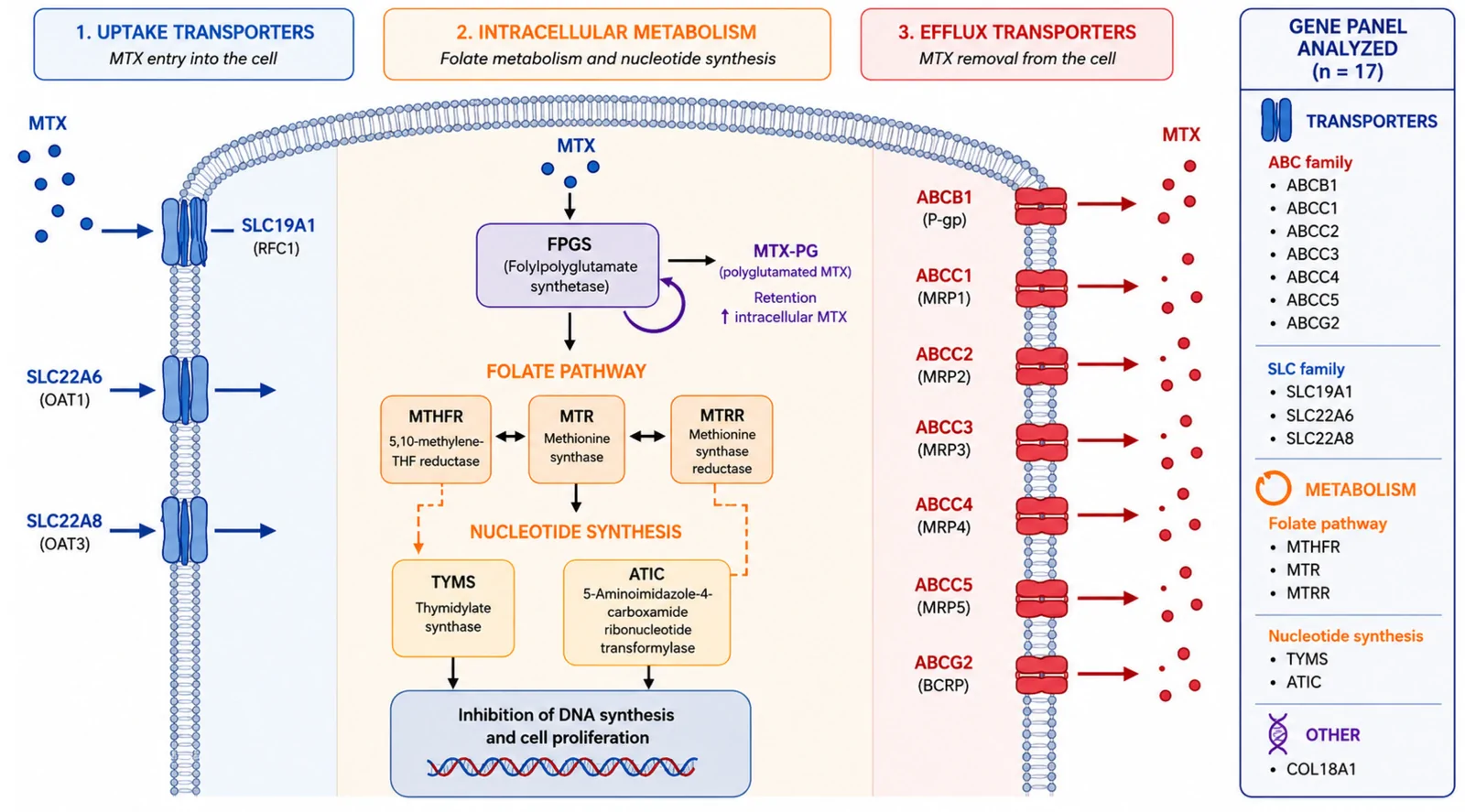
Schematic overview of MTX transport, intracellular metabolism, and targeted gene panel analyzed in this study. Schematic representation of MTX cellular transport, intracellular metabolism, and efflux mechanisms. MTX enters the cell primarily via solute carrier transporters, undergoes intracellular polyglutamation mediated by *FPGS*, and participates in folate metabolism and nucleotide synthesis pathways, ultimately leading to inhibition of DNA synthesis and cell proliferation. MTX is exported from the cell through ATP-binding cassette transporters. The figure highlights the 17 genes included in the targeted sequencing panel. ABC, ATP-binding cassette, MTX, methotrexate.

MTX intracellular routes are regulated by a complex network of transporters and folate-dependent enzymes. Based on this biological framework, a targeted next-generation sequencing (NGS) panel was designed to analyze 17 genes involved in MTX cellular efflux/uptake mediated by drug transporters, intracellular polyglutamation and folate-dependent metabolism (Figure 1). The panel included the coding DNA sequence and UTR regions of solute carrier transporters mediating MTX uptake (*SLC19A1*, *SLC22A6*, *SLC22A8*), ATP-binding cassette transporters responsible for drug efflux (*ABCB1*, *ABCC1–5*, *ABCG2*), and enzymes involved in intracellular MTX metabolism and folate-dependent pathways (*FPGS*, *MTHFR*, *MTR*, *MTRR*, *TYMS*, and *ATIC*). Additionally, *COL18A1* was included due to its potential role in extracellular matrix organization and vascular integrity, which may influence blood–brain barrier function and susceptibility to NTX. *COL18A1* was considered a biologically plausible candidate for exploratory pharmacogenetic analysis, despite not being directly involved in MTX transport or folate metabolism.

### 2.3. DNA Extraction and Targeted Sequencing

Peripheral blood samples were obtained from all patients and genomic DNA purified using a standard phenol–chloroform extraction with ethanol precipitation procedure. Extracted DNA was quantified using a Qubit™ dsDNA BR Assay Kit on a Qubit 2.0 Fluorimeter (Thermo Fisher Scientific, Inc. – Waltham, Massachusetts, USA), following the manufacturer’s instructions, while DNA purity was assessed using Nanodrop by measuring absorbance ratios. The A260/A280 and A260/A230 ratios were found to be within the 1.8 to 2.0 range, indicating adequate DNA purity for downstream molecular analyses. DNA samples meeting these quality criteria were subsequently used for multiplex PCR and targeted next-generation sequencing. No additional DNA fragmentation assessment was performed prior to library preparation.

Manual library preparations were performed using an Ion AmpliSeq™ Custom Panel, IonCode™ Barcode Adapters 1-96 Kit and an Ion AmpliSeq Library Kit 2.0 (Thermo Fisher Scientific, Inc.). The panel consisted of 736 amplicons of approximately 200 bp each. In silico coverage was 99.11% (missed bp: 829). Multiplex PCR was performed using 10 ng genomic DNA with a premixed primer pool and Ion AmpliSeq HiFi master mix (Ion AmpliSeq Library Kit 2.0). The amplicons were treated with 2 µL FuPa reagent to partially digest the primer sequences and phosphorylate the amplicons. Amplicons were ligated to adapters with the barcodes of IonCode™ Barcode Adapters kit (Thermo Fisher Scientific, Inc.). The adapter-ligated amplicons (library) were purified using the Agencourt AMPure XP reagent (Beckman Coulter, Inc. – Brea, California, USA). Quantification of the final libraries was performed using an Ion Library TaqMan™ Quantitation Kit (Thermo Fisher Scientific, Inc.) on the Applied Biosystems^®^ QuantStudio™ 6 Flex Real-Time PCR System, following the manufacturer’s protocol.

After library dilution to 100 pM, the clonal amplification of barcoded DNA library (AmpliSeq libraries) onto ion spheres was performed on an Ion Chef™ Instrument using Ion 510 & Ion 520 & Ion 530 Kit-Chef, according to the manufacturer’s instructions (Thermo Fisher Scientific, Inc.). Template-positive spheres from barcoded libraries were multiplexed and loaded onto Ion 530™ Chips following the manufacturer’s protocol, and sequencing was run on the Ion Gene Studio S5 XL system (Thermo Fisher Scientific, Inc.). Sequencing quality was assessed using the Ion Torrent Coverage Analysis workflow. The targeted sequencing approach provided an expected mean read depth of up to approximately 1500×, with an in silico target region coverage of 99.11%. A water-only negative control was included during library preparation to monitor for potential contamination and nonspecific amplification. No external standard control samples with predefined variants were included for independent validation of variant accuracy.

### 2.4. Bioinformatic Analysis

In the reported sequencing run, the mean target depth was approximately 2000×, with 99.37% on-target reads and 95.93% mean coverage uniformity. Raw sequencing data were processed using Torrent Suite version 5.12.2. The run configuration included quality filtering, a base-quality trimming threshold of 15, a minimum barcode read threshold of 10 reads, and phasing residual filtering with a threshold of 2.0. Sequencing reads were aligned against the human reference genome hg19 (GRCh37), and variant annotation was performed using the Ensembl Variant Effect Predictor (VEP; version 114). Annotated variants from all patients were imported into R for downstream bioinformatic analyses. No predefined minor allele frequency (MAF) threshold or Hardy–Weinberg equilibrium (HWE) filtering was applied before the association analysis. Variants were filtered to retain only those mapping to the genes included in the study panel and corresponding to curated RefSeq transcripts (NM identifiers). In cases where multiple transcript versions were available, the highest transcript version was selected to ensure annotation consistency. Duplicate variants within the same patient were removed, generating a non-redundant variant dataset for subsequent analyses.

A binary genetic matrix representing the presence or absence of each variant in every individual was subsequently generated based on variant carrier status. Both heterozygous and homozygous alternate genotypes were coded as 1 (variant present), whereas individuals without the variant were coded as 0. Variant identifiers were constructed using gene symbol and HGVS coding nomenclature to facilitate downstream association and burden analyses. Following filtering and transcript harmonization, a high-confidence dataset was obtained for statistical analyses and visualization.

### 2.5. Statistical Analysis

Comparisons between patients with and without MTX-associated neurotoxicity were performed using Fisher’s exact test. To control for multiple testing, false discovery rate (FDR) correction was applied separately to the overall MTX-NTX and SLS analysis, using the Benjamini–Hochberg method. Significant genetic associations were further evaluated using multivariable Firth penalized logistic regression adjusted for age and sex. Firth regression was selected to reduce small-sample bias and address potential separation associated with low-frequency variants. Given the limited number of MTX-NTX events, the number of covariates was deliberately restricted to minimize model overfitting. Odds ratios (ORs) with 95% confidence intervals (95% CIs) were calculated to estimate the magnitude and direction of association between genetic variants and MTX-NTX susceptibility. Clinical phenotypes related to MTX-NTX were manually reviewed and encoded as binary variables. In addition, specific neurological manifestations were independently categorized for phenotype-oriented analyses. Both variant-level and gene-level exploratory burden analyses were performed. Given the high sequencing depth achieved (mean coverage approximately 2000×), candidate variant calls were additionally reviewed manually using the Integrative Genomics Viewer (IGV) to assess the distribution and consistency of the reads supporting each variant and to identify potential mapping or sequencing artefacts. No orthogonal validation was performed.

Given that SLS represented the most frequent neurological phenotype, all analyses were subsequently replicated in the SLS subgroup to evaluate the consistency of the observed associations. Pathway-oriented analyses were conducted by comparing the cumulative burden of variants involved in MTX transport and folate/metabolism pathways between groups using Wilcoxon rank-sum tests. To compare the genetic architecture of overall MTX-associated neurotoxicity and the SLS phenotype, the distribution of significant variants across genes was assessed using Spearman’s rank correlation coefficient. Additional analyses included phenotype-specific burden analyses evaluating the distribution of variants according to individual neurological manifestations. For the purpose of variant quantification, a distinction was made between variant observations and unique genomic variants. A variant observation was defined as the presence of a given genomic variant in an individual patient. Unique variants were defined as distinct genomic variants identified across the entire study cohort, irrespective of the number of patients carrying them.

Graphical representations included volcano plots, heatmaps, correlation matrices, UpSet plots, and circular packing visualizations to explore variant distribution patterns, phenotype overlap, and gene-level burden profiles. Statistical analyses and data visualization were performed using R statistical software (version R 4.1.3) with packages *tidyverse*, *ggplot2*, *broom*, *pheatmap*, *corrplot*, *UpSetR*, *patchwork*, and related dependencies.

## 3. Results

### 3.1. Baseline and Clinical Characteristics of Neurotoxicity Events

A total of 34 pediatric patients with MTX-NTX were included in the study whose mean age at diagnosis was 10.7 ± 3.9 years, compared with 6.7 ± 4.5 years in the control group (*p* < 0.001). It should be remarked, however, that the control group consisted of randomly selected patients representing the average age at leukemia diagnosis, and it is known that NTX occurs in older patients [9]. Regarding treatment protocols, most patients were treated according to the SEHOP-PETHEMA 2013 protocol (55.9%), as it occurred in the children without NTX (51.4%, *p* = 0.523). Neurotoxicity events occurred most frequently during the consolidation phase and SLS was the most frequent clinical phenotype, accounting for 58.8% of neurotoxicity events, whilst isolated seizures were uncommon, representing only one case. All these and other characteristics of the cohort are shown in Table 1. The distribution of treatment protocols and the cumulative MTX exposure showed no statistically significant differences between groups. The mean cumulative IV MTX dose received was 22.0 ± 12.22 g/m^2^ in the neurotoxicity group compared to 22.95 ± 12 g/m^2^ in controls (*p* = 0.743). Similarly, the mean number of IT MTX doses administered was 11.35 ± 4.38 in NTX cases versus 12.73 ± 2.75 in controls (*p* = 0.114). The mean interval between MTX administration and neurotoxicity onset was 8.8 ± 4.6 days, and most events (91.2%) occurred within the first two weeks after MTX exposure.

### 3.2. Distribution of Genetic Variants in MTX-Related Genes

**Figure 2:**
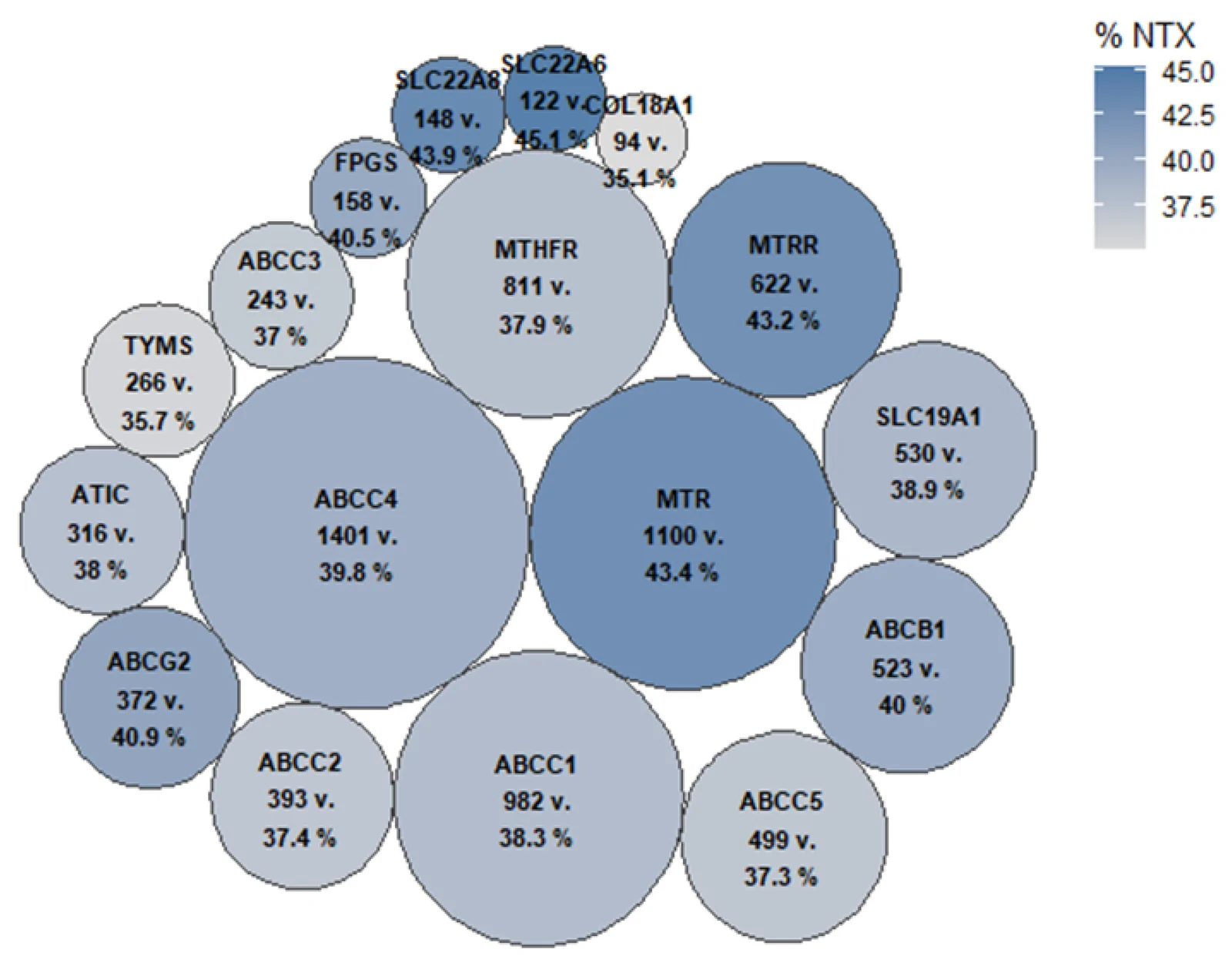
Global distribution of genetic variants in MTX-related genes in patients with neurotoxicity. Bubble plot represents the total number of variants identified in each gene (circle size) and the percentage of patients with neurotoxicity carrying variants in each gene (color intensity). NTX, neurotoxicity; v., variants.

The global distribution of genetic variants across MTX-related genes is shown in Figure 2. Across all patients, 8486 variant observations were identified, corresponding to 2575 unique genomic variants. Here, variant observations refer to the presence of a given variant in an individual patient, whereas unique variants represent distinct genomic variants identified across the entire cohort. *ABCC4* showed the greatest number of variants overall (*n* = 1401), corresponding to 252 unique variants, followed by *MTR* (1100; 298 unique variants) and *ABCC1* (982; 258 unique variants). Transporter-related genes accounted for 5213 observations (61.4%), whereas folate metabolism and intracellular MTX metabolism genes accounted for 3273 (38.6%). *SLC22A6*, *SLC22A8*, *MTR* and *MTRR* were the genes with the highest percentage of variants present in the MTX-NTX group (45.1, 43.9, 43.4 and 43.2%, respectively).

As SLS was the main clinical phenotype, subsequent analyses were carried out both in the whole MTX-NTX group and in the SLS subset.

### 3.3. Pathway-Level Burden Analysis

**Figure 3:**
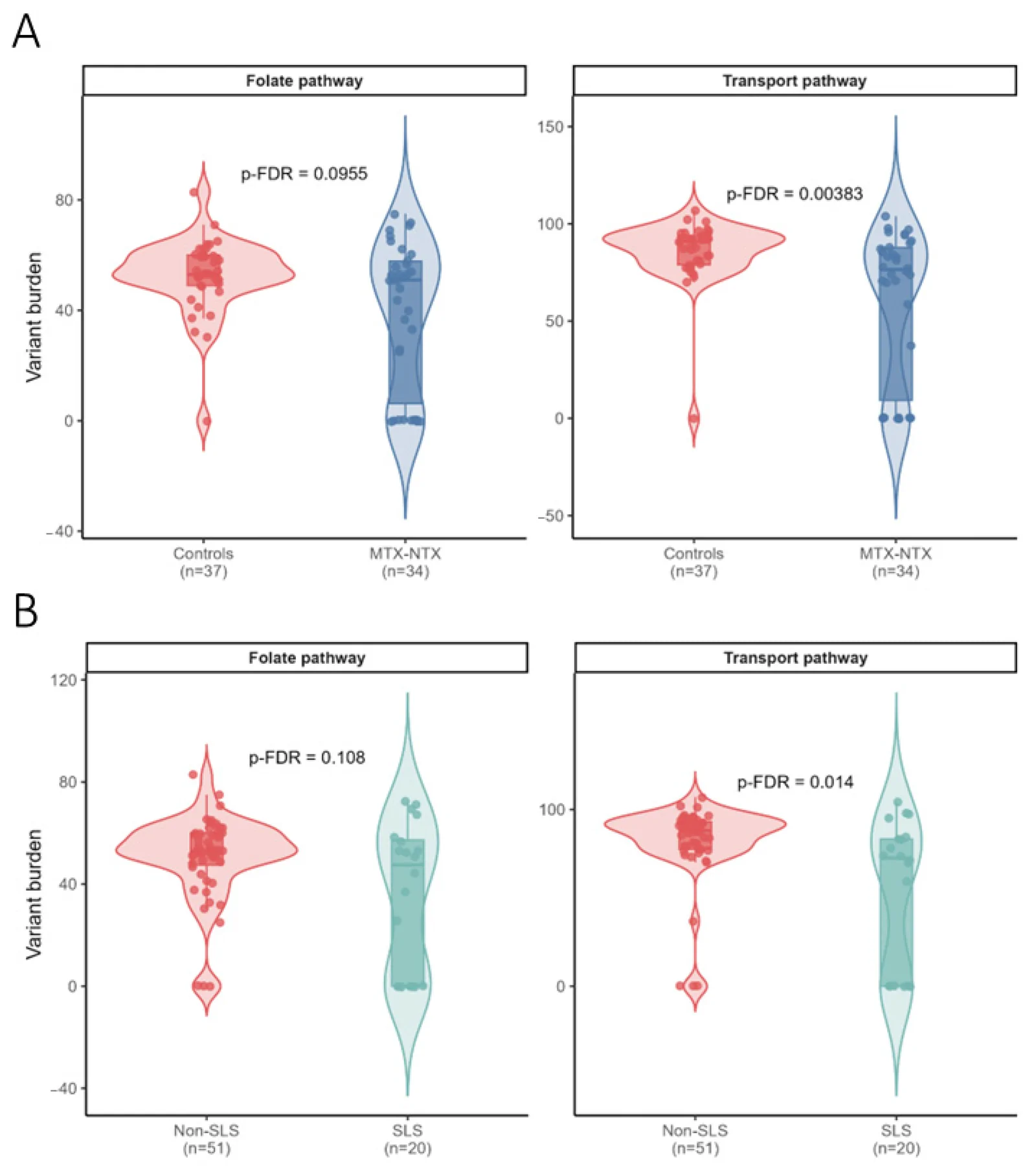
Pathway-level burden analysis for MTX-induced neurotoxicity. (**A**) Distribution of the number of variants in genes related to MTX transport and folate metabolism comparing control subjects (*n* = 37; red) and MTX-NTX patients (*n* = 34; blue). (**B**) Distribution of the number of variants burden in the same pathways, comparing non-SLS (*n* = 50; red) and SLS (*n* = 20; green) patients. Data are presented as violin-box plots showing the median, interquartile range, and individual data points. FDR-adjusted *p* values are indicated for significant differences between cohorts. MTX-NTX, methotrexate-induced neurotoxicity; SLS, stroke-like syndrome.

Pathway-level burden analyses were performed comparing the total number of variants identified in MTX transporter- and folate/metabolism-related genes between cases and controls. Patients with MTX-NTX showed a significantly different burden distribution of transporter-related variants compared with controls (FDR-adjusted *p* = 0.004, Figure 3A). In contrast, the difference for folate/metabolism-related variants did not remain significant after multiple-testing correction (FDR-adjusted *p* = 0.096, Figure 3A). Similar findings were observed for the SLS subgroup (FDR-adjusted *p* = 0.014 and FDR-adjusted *p* = 0.108 for transporter- and folate-related genes, respectively, Figure 3B).

### 3.4. Gene-Level Burden Analysis Across Neurotoxicity Phenotypes

**Figure 4:**
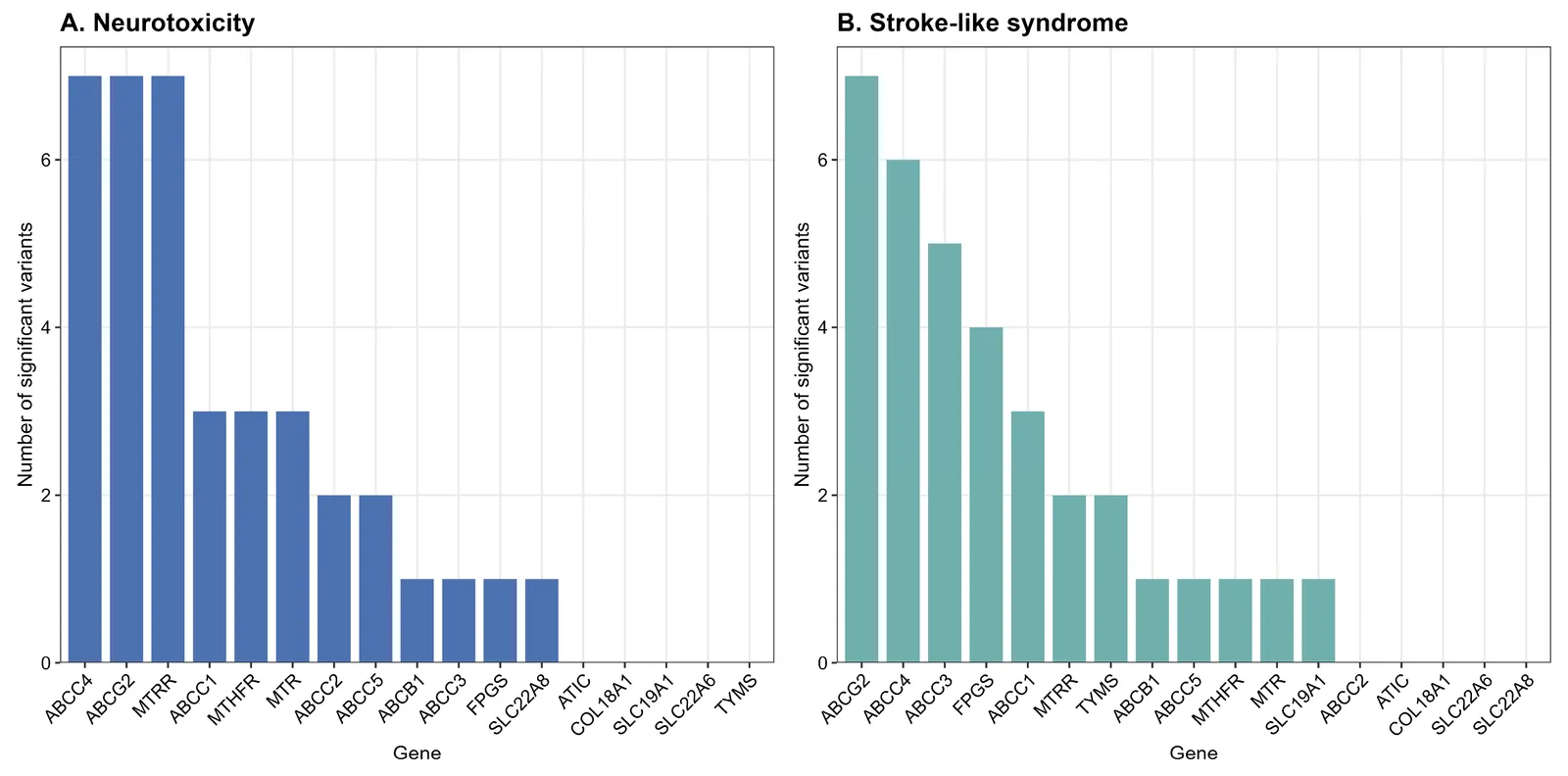
Distribution of significant genetic variants across genes in MTX-induced neurotoxicity. (**A**) Number of significant variants identified in each gene in the global neurotoxicity analysis. (**B**) Number of significant variants identified in the stroke-like phenotype subgroup. Bars represent the total number of significant variants per gene. Genes are displayed on the x-axis and the number of significant variants on the y-axis.

As in the pathway-level analysis, a similar pattern of affected genes was observed for both the MTX-NTX and SLS groups. *ABCG2* and *ABCC4* were the genes with the highest number of nominally significant variants regarding MTX-NTX risk, with both 7 out of a total of 38 (18.4%), and 7/34 (20.6%) and 6/34 (17.6%) respectively, for SLS (Figure 4). In the MTX-NTX group *MTRR* (18.4%) was another relevant loci, whereas *ABCC3* and *FPGS* showed a greater contribution in the SLS phenotype (5/34, 14.7% and 4/34, 11.8%, respectively). Notably, the three most frequently represented genes in the SLS group (*ABCG2*, *ABCC4*, and *ABCC3*) accounted for 52.9% of all variants identified in this phenotype. A significant positive correlation was observed between the distribution of variants in the studied genes between the MTX-NTX and SLS groups (Spearman’s ρ = 0.549, *p* = 0.022), indicating that genes enriched in the global neurotoxicity analysis also tended to be enriched in patients with the SLS phenotype.

### 3.5. Association Analysis of Significant Variants

**Figure 5:**
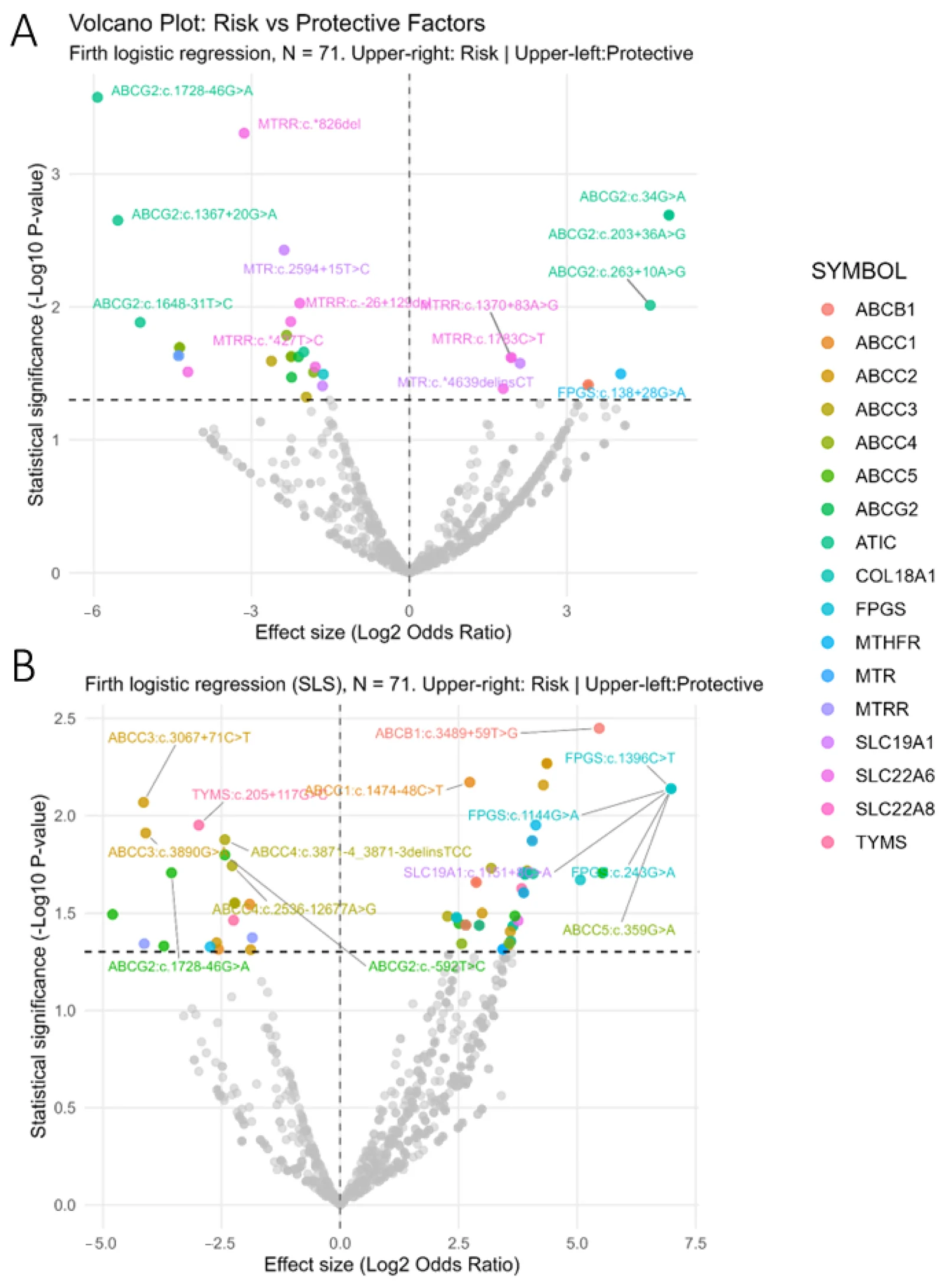
Association analysis of genetic variants using volcano plots. (**A**) Global analysis showing the relationship between effect size (log2 odds ratio) and statistical significance (−log10 *p*-value) for each variant. Variants above the dashed horizontal line meet the predefined significance threshold. Variants on the right indicate increased risk, while those on the left indicate protective effects. (**B**) Analysis restricted to the stroke-like (SLS) phenotype subgroup. Each point represents an individual variant, labeled for the most significant associations and colored according to the corresponding gene. The asterisk (*) in variant nomenclature (e.g., MTRR:c.*826del) indicates a position downstream of the translation termination codon, within the 3′ untranslated region (3′ UTR).

Volcano plot analyses enabled the identification of specific genetic variants associated with risk of NTX. In the global analysis (Figure 5A), a total of 38 nominally statistically significant variants were detected, of which 26 (68.4%) showed protective associations and 12 (31.6%) were associated with increased risk. The variants showing the largest negative effect sizes were located in a transporter gene, namely *ABCG2* [c.1728-46G>A (OR = 0.016, *p* = 2.6 × 10^−4^) and c.1367+20G>A (OR = 0.02, *p* = 0.0022)]. In the same manner, the three most prominent risk variants were located in *ABCG2* [c.203+36A>G (OR = 30.76, *p* = 0.002), c.34G>A (OR = 30.76, *p* = 0.002), and c.263+10A>G (OR = 24.01, *p* = 0.009)].

Within the SLS subgroup (Figure 5B), *ABCG2* still showed a prominent role, with two of the variants displaying nominally significant protective associations, namely c.1728-46G>A (OR = 0.084, *p* = 0.019) and c.-592T>C (OR = 0.185, *p* = 0.015). Conversely, among the variants associated with increased SLS risk, three variants in *FPGS* were identified (c.243 G>A, c.1144G>A and c.1396C>T; OR = 12.62, *p* = 0.0073).

Among the significant variants identified in both groups, several were detected exclusively in either cases or controls. In the overall NTX group, all of these (5) were risk variants and located in ABC genes, three of them in *ABCG2*: c.203+36A>G (*p* = 0.002), c.34G>A (*p* = 0.002) and c.263+10A>G (*p* = 0.009) (Supplementary Table S1). In the SLS group, the most remarkable finding was that *TYMS* c.*89A>G and c.205+117G>C were found in 20 and 17 controls (*p* = 0.0001 and *p* = 0.002, respectively), but no patients with NTX carried these mutations (Supplementary Table S1).

### 3.6. Symptom-Specific Genetic Architecture of MTX-Induced Neurotoxicity

**Figure 6:**
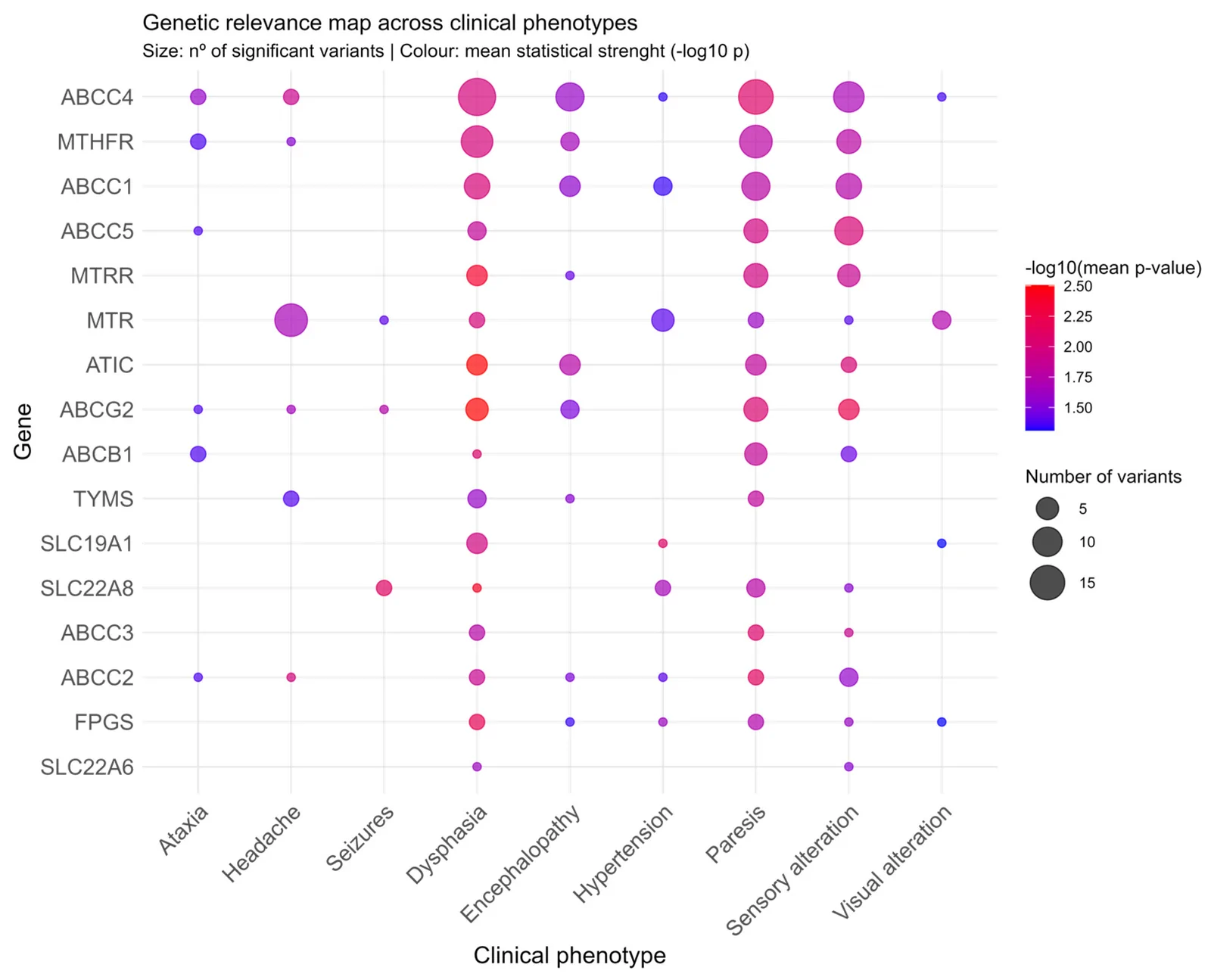
Symptom-specific genetic relevance map of MTX-induced neurotoxicity showing the distribution of significant genetic variants across genes and clinical phenotypes. Circle size represents the number of significant variants identified in each gene for a given phenotype, while color intensity reflects the average statistical significance (−log10 *p*-value).

We next investigated symptom-specific genetic signatures. A total of 282 associations (153 unique variants) showed nominal statistical significance (*p* < 0.05) across the different neurological manifestations. The highest number of nominally significant variants was observed for paresis (*n* = 77) and dysarthria/dysphasia (*n* = 71), whilst seizures showed the lowest (*n* = 4). *ABCC4* was the most represented gene in the recorded symptoms, with 22 nominally significant variants (Figure 6).

Overall, transporter genes accounted for 61.4% of the nominally significant variants, whilst folate/metabolism-related genes accounted for 38.6%. However, the relative contribution of these two biological pathways varied significantly among clinical symptoms (χ^2^ = 21.5, *p* = 0.006). Ataxia, paresis, dysarthria/dysphasia, and sensory disturbances showed a clear predominance of transporter-related mutations, accounting for 77.8, 63.3, 62.0, and 72.2% of nominally significant variants, respectively.

## 4. Discussion

The etiopathogenesis of MTX-NTX in children with ALL is still poorly understood, and a variety of clinical and demographic risk factors have been proposed [9,11,23,24]. In addition, in recent years, special emphasis has been placed on the role of MTX-related genes [9,15,25]. The present study provides a comprehensive characterization of the pharmacogenetic architecture underlying MTX-NTX through targeted NGS of transport- and folate-related genes in a multicenter nationwide pediatric ALL cohort.

The most relevant observation emerging from our analyses was the predominant contribution of ABC transporters, particularly *ABCC4*, *ABCG2*, *ABCB1*, and *ABCC1*, across pathway-level analyses, burden analyses, volcano plot associations, and phenotype-specific analyses. MTX disposition depends on a coordinated network of influx and efflux transporters regulating intracellular drug accumulation and blood–brain barrier permeability. Experimental models have demonstrated that impaired ABC transporter activity significantly modifies MTX PK and tissue exposure, providing a biologically plausible explanation for our findings [10,17,26]. However, there are also studies that argue against a link between MTX PK and MTX-NTX [9]. Indeed, our findings should also be interpreted in the context of previous genome-wide studies, which suggest that susceptibility to MTX-NTX extends beyond classical MTX pharmacokinetic pathways. A GWAS of MTX-NTX in childhood ALL pinpointed variants near genes involved in neuronal growth, differentiation, and cytoskeletal organization rather than classic PK genes [9]. Therefore, other hypotheses supporting the involvement of ABC transporters in MTX-NTX must be considered—for instance, neuroinflammation, as these transporters also regulate immune-cell trafficking and cytokine milieu at the blood–brain barrier [27]. In addition, ABC genes can also transport endogenous metabolites and nucleotides [28,29], and the alteration of intra-CNS levels of MTX metabolites could therefore amplify neurotoxic cascades without changing systemic MTX PK. These findings are not necessarily contradictory but rather suggest complementary mechanisms of susceptibility.

Among these transporters, *ABCG2*, coding for the breast cancer resistance protein (BCRP), showed the most prominent pattern of associations in our analysis. The volcano plot showed that two *ABCG2* variants clustered in the upper-left quadrant, suggesting nominally significant associations in the protective direction, whilst three additional variants showed the strongest associations with increased risk of MTX-NTX. Furthermore, these three risk variants [c.34G>A (rs2231137), c.203+36A>G (rs4148152), and c.263+10A>G (rs2231138)] were detected exclusively in patients with MTX-NTX. Previous studies have shown the importance of the ABC transporter gene loci for MTX-NTX in childhood ALL [16]. In particular, the identified missense variant *ABCG2* rs2231137 has previously been associated with reduced transporter activity and altered MTX PK, suggesting that diminished BCRP-mediated efflux may increase CNS exposure to MTX and promote neurological toxicity [30,31]. Likewise, although the functional impact of the intronic variants remains uncertain, previous studies have suggested that regulatory *ABCG2* polymorphisms may influence transporter expression and substrate handling [32,33]. Taken together, these findings identify *ABCG2* as a potential candidate for further investigation in MTX-NTX while functional studies and replication in larger, independent cohorts are needed to determine whether these variants contribute directly to MTX-related neurotoxicity.

Our pathway-level burden analysis showed that overall, variants affecting folate metabolism appeared to have a lower contribution to the development of MTX-NTX, although *MTHFR*, *MTR*, *MTRR*, and *TYMS* still exhibited a substantial burden of significant variants. Previous pharmacogenetic studies evaluating variability in these genes have mostly reported controversial associations with MTX toxicity [16,25,34,35], although most of the studies lacked an integrative approach and analyzed the SNPs individually.

From a clinical perspective, SLS represented the predominant neurological phenotype, in our patients, in agreement with what has been described for contemporary pediatric cohorts [12,17,36]. Gene-level burden analysis showed a remarkable similarity between the genetic architecture of global MTX-NTX and the SLS phenotype. Indeed, most of the variants highlighted in the volcano plot for NTX, e.g., *ABCG2* 1728-46G>A, *ABCG2* 1367+20G>A, or *ABCB1* 3489+59T>G, also stood out in the SLS subgroup. This would suggest that SLS represents the predominant clinical expression of a common pharmacogenetic susceptibility rather than a biologically distinct entity. If anything, a slightly higher contribution of folate-related variants was observed in the SLS subgroup, with two SNPs in *TYMS* displaying very relevant protective ORs. *TYMS* encodes thymidylate synthase, one of the principal intracellular targets of MTX, and regulatory variants affecting *TYMS* have previously been associated with differences in MTX-related toxicity [37]. In particular, *TYMS* c.*89A>G (rs2790) was exclusively found in children without NTX in our study. One meta-analysis has reported this SNP to be associated with total cancer risk, although only two studies have analyzed it in leukemia patients. Wang et al. observed an association with altered MTX blood levels in children with ALL [38]; in contrast, Al-Sheikh et al. could not find a link to hematological toxicities. Interestingly, the SLS-specific analysis identified three nominally significant variants in *FPGS* (c.243G>A, c.1144G>A, and c.1396C>T), which were more frequently observed among cases than controls. *FPGS* catalyzes the polyglutamylation of methotrexate, thereby contributing to the pharmacodynamic effects. Previous studies have linked *FPGS* variation, including c.1396C>T (rs35789560), to altered methotrexate polyglutamate accumulation and treatment-related outcomes [39]. These findings suggest that *FPGS* variation may contribute to susceptibility to specific neurological manifestations of MTX toxicity, although this association requires validation in larger independent cohorts and functional studies. Last, but not least, two other pinpointed protective variants in the SLS group, *ABCC1* c.*801G>C (rs129081) and c.3079+62T>C (rs3887893), have previously been associated with interindividual differences in MTX PK and treatment response [19,40,41,42].

The phenotype-specific analyses suggest that MTX-NTX may encompass heterogeneous clinical manifestations with potentially different patterns of pharmacogenetic associations. Transporter-related genes predominated overall, especially in focal manifestations such as paresis, dysarthria/dysphasia, ataxia, and sensory disturbances. This pattern is biologically plausible given the involvement of MTX transporters in drug distribution and intracellular accumulation [22]. In contrast, headache and visual disturbances exhibited a greater contribution from folate metabolism-related genes, although these observations should also be interpreted cautiously. In particular, some symptom-specific subgroups were very small, including ataxia (*n* = 3) and isolated seizures (*n* = 1), substantially limiting statistical power and the reliability of variant-level associations. Therefore, these phenotype-specific findings should be considered exploratory and hypothesis-generating, and require replication in larger, adequately powered cohorts before specific genotype–phenotype or biological relationships can be inferred.

From a translational perspective, these findings may contribute to future individualized MTX management. Previous pharmacogenomic studies have shown that interpatient variability in MTX disposition and intracellular accumulation is influenced by treatment, pharmacokinetic, and genetic factors, including MTX transporters such as *ABCC1* and *ABCC4* [18]. If prospectively validated, the genetic signatures identified here could complement clinical and pharmacokinetic parameters to identify patients at increased risk of MTX-NTX, supporting closer neurological monitoring and, ultimately, more individualized therapeutic strategies. However, the present findings remain exploratory and do not currently support genotype-guided MTX dose modification [17,22].

Several strengths of the present study should be highlighted. First, this represents a nationwide multicenter cohort including patients from multiple pediatric oncology centers across Spain, providing one of the largest genetically characterized series of MTX-NTX reported to date, despite the relatively low incidence of both childhood ALL and MTX-NTX. Second, the targeted sequencing strategy was intentionally restricted to candidate genes involved in MTX transport and folate metabolism according to the biological evidence. This candidate gene approach enabled deep sequencing of predefined biologically relevant regions and facilitated the detailed characterization of genetic variation across complementary pharmacological pathways. Third, the study incorporated a detailed phenotypic characterization of MTX-NTX rather than treating neurotoxicity as a single homogeneous outcome. Finally, the analytical strategy integrated variant-level association testing with gene-level, pathway-level, and phenotype-specific analyses. This multidimensional approach allowed the identification of recurrent patterns across complementary analytical levels and provided a framework for generating testable hypotheses regarding the contribution of transporter and folate-related pathways to MTX-NTX.

Our study has several limitations that should be acknowledged when interpreting the present findings. The relatively limited sample size limits statistical power for the detection of rare variants and precluded the use of multivariate analyses adjusting for clinical covariates. While this approach maximizes sequencing depth and analytical sensitivity for biologically relevant pathways, it does not allow the identification of novel susceptibility loci outside the selected regions and therefore cannot provide an unbiased genome-wide assessment of genetic risk. Future genome-wide approaches and functional validation studies will therefore be necessary to expand the current pharmacogenetic landscape of MTX-NTX. The present candidate-gene findings should therefore be considered complementary to, rather than a substitute. No predefined minor allele frequency threshold was applied, as we aimed to retain potentially informative rare variants in this exploratory study. The statistical associations identified in transporter-related genes, particularly *ABCC4* and *ABCG2*, should not be interpreted as evidence of biological causality. No functional experiments were performed to determine whether the identified variants modify transporter expression or activity, methotrexate disposition, or mechanisms directly involved in neurological injury. Similarly, the findings were not replicated in an independent cohort. Consequently, the biological and clinical relevance of the identified variants remains to be established. Finally, future prospective multicenter studies incorporating larger sample sizes, genome-wide approaches, functional characterization, and independent replication will be required to confirm and extend the present findings.

## 5. Conclusions

MTX neurotoxicity does not appear to be a single entity but rather a spectrum of phenotypes with partially distinct pharmacogenetic architectures. Furthermore, our findings suggest that genetic variability in ABC transporter genes may contribute substantially to susceptibility to MTX-NTX, whereas folate metabolism genes may act as complementary phenotype-modifying factors. The recurrent implication of *ABCC4*, *ABCG2*, *ABCB1*, *ABCC1*, *MTHFR*, *MTR*, *MTRR*, *TYMS* and *FPGS* across multiple independent analyses highlights these genes as potential candidates for future pharmacogenomic investigation. However, their clinical predictive value remains unestablished, requiring rigorous validation in larger prospective multicenter cohorts, together with functional characterization of the identified variants. This may enable the implementation of individualized MTX treatment strategies aimed at minimizing neurotoxicity while preserving antileukemic efficacy.

## Figures and Tables

**Table 1:** Baseline characteristics of participants that developed MTX-related neurotoxicity.

Characteristics	MTX-NTX	Controls	*p*-Value
Age at diagnosis, years (mean ± SD)	10.7 ± 3.9	6.7 ± 4.5	*p* < 0.001
Age < 10 years	9 (26.5%)	28 (75.7%)	*p* < 0.001
Males	15 (44.1%)	14 (37.8%)	*p* = 0.65
Lineage			*p* = 0.685
B-lineage	28 (82.4%)	27 (73.0%)	
T-lineage	5 (14.7%)	9 (24.3%)	
Biphenotypic	1 (2.9%)	1 (2.7%)	
Treatment protocol			*p* = 0.936
LAL-SHOP-2005	12 (35.3%)	15 (40.5%)	
SEHOP-PETHEMA 2013	19 (55.9%)	19 (51.4%)	
LAL-SHOP-99	3 (8.8%)	3 (8.1%)	
Treatment phase in which NTX originated			
Consolidation	17 (50.0%)		
Induction	9 (26.5%)		
Maintenance	3 (8.8%)		
Reinduction	2 (5.9%)		
Intensification	2 (5.9%)		
Other	1 (2.9%)		
Administration of MTX that led to NTX			
Intrathecal	15 (44.1%)		
Intrathecal + Intravenous	19 (55.9%)		
Clinical phenotype			
SLS	20 (58.8%)		
PRES	6 (17.6%)		
MTX-NTX NOS	7 (20.6%)		
Seizures (isolated)	1 (2.9%)		
Neurological manifestations			
Dysphasia	20 (58.8%)		
Paresis	19 (55.9%)		
Encephalopathy	16 (47.1%)		
Headache	11 (32.4%)		
Seizures	10 (29.4%)		
Sensory disturbances	10 (29.4%)		
Hypertension	7 (20.6%)		
Visual disturbances	6 (17.6%)		
Ataxia	3 (8.8%)		

NTX, neurotoxicity; MTX, methotrexate; MTX-NTX, methotrexate-induced neurotoxicity; SLS, stroke-like syndrome; PRES, posterior reversible encephalopathy syndrome; MTX-NTX NOS, methotrexate-induced neurotoxicity not otherwise specified.

## Data Availability

The anonymized datasets generated and analyzed during the current study have been deposited in the Figshare repository and are publicly available at https://doi.org/10.6084/m9.figshare.32851256. The repository includes anonymized clinical data and variant call format (VCF) files generated from the targeted next-generation sequencing panel.

## Supplementary Materials

The following supporting information can be downloaded at https://www.mdpi.com/article/10.3390/jcm15176877/s1: Table S1: Group-exclusive variants associated with MTX-induced neurotoxicity phenotypes.

## Abbreviations

The following abbreviations are used in this manuscript:

MTX	Methotrexate
MTX-NTX	Methotrexate-induced neurotoxicity
ALL	Acute lymphoblastic leukemia
ABC	ATP-binding cassette
NGS	Next-generation sequencing
MTX-NTX-NOS	MTX-induced neurotoxicity not otherwise specified
SLS	Stroke-like syndrome
PRES	Posterior reversible encephalopathy syndrome
CNS	Central Nervous System
PK	Pharmacokinetics
SEHOP	Spanish Society of Pediatric Hematology and Oncology
