# Supplementary material for: Phenotype-Specific Pharmacogenetic Patterns of Methotrexate Neurotoxicity in Pediatric Acute Lymphoblastic Leukemia
Source: J Clin Med. 2026 Sep 5;15(17):6877. doi: 10.3390/jcm15176877 (PMC13565789; doi:10.3390/jcm15176877)
Supplement: Supplementary file 1 [file jcm-15-06877-s001.zip › jcm-4487245-supplementary.pdf]

Table S1. Group-exclusive variants associated with MTX-induced neurotoxicity phenotypes

Group	Gene	Variant	Consequence	Cases	Controls	p-value	FDR-p	Effect
NTX	ABCG2	c.203+36A>G	Intron variant	8	0	0.0017	0.048	R
NTX	ABCG2	c.34G>A	Missense variant	8	0	0.0017	0.048	R
NTX	ABCG2	c.263+10A>G	Intron variant	6	0	0.0094	0.134	R
NTX	ABCB1	c.3489+59T>G	Intron variant	4	0	0.0477	0.330	R
NTX	ABCC4	c.3211-39_ 3211-35del	Intron variant	4	0	0.0477	0.330	R
SLS	ABCB1	c.3489+59T>G	Intron variant	4	0	0.0050	0.091	R
SLS	ABCC4	c.3211-39_3211-35del	Intron variant	4	0	0.0050	0.091	R
SLS	MTRR	c.903+518T>A	Intron variant	3	0	0.0199	0.204	R
SLS	TYMS	c.*89A>G	3′ UTR variant	0	20	<0.0001	0.057	P
SLS	TYMS	c.205+117G>C	Intron variant	0	17	0.0017	0.075	P
SLS	ABCG2	c.-490C>G	5′ UTR variant	0	15	0.0073	0.105	P
SLS	ABCC3	c.3067+71C>T	Intron variant	0	13	0.0141	0.162	P
SLS	ABCC3	c.*768C>T	3′ UTR variant	0	12	0.0152	0.162	P

NTX, methotrexate-induced neurotoxicity; SLS, stroke-like syndrome; R, risk; P, protective.
